# Supplementary material for: Caspase-Mediated Regulation and Cellular Heterogeneity of the cGAS/STING Pathway in Kaposi’s Sarcoma-Associated Herpesvirus Infection
Source: mBio. 2022 Oct 18;13(6):e02446-22. doi: 10.1128/mbio.02446-22 (PMC9765453; doi:10.1128/mbio.02446-22)
Supplement: FIG S1 [file mbio.02446-22-sf001.pdf]

Figure S1

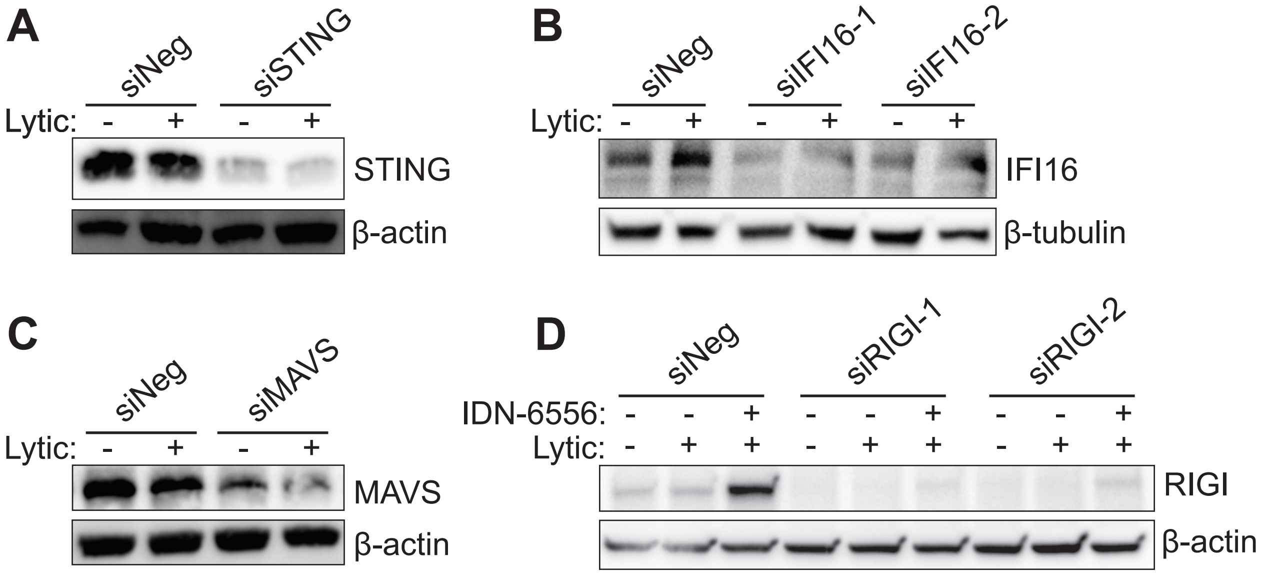

Figure S1. Knockdown of proteins in the type I IFN induction pathway.
iSLK.219 cells were transfected with a negative control siRNA and siRNAs targeting STING (A), IFI16 (B), MAVS (C), or RIG-I (D). Cell lysates were harvested at day 4 post reactivation with doxycycline and treatment with IDN-6556 (where indicated) and subjected to western blotting for the target proteins and β-actin or β-tubulin as loading controls. Blots are representative of 3 replicates.
