# Supplementary material for: Caspase-Mediated Regulation and Cellular Heterogeneity of the cGAS/STING Pathway in Kaposi’s Sarcoma-Associated Herpesvirus Infection
Source: mBio. 2022 Oct 18;13(6):e02446-22. doi: 10.1128/mbio.02446-22 (PMC9765453; doi:10.1128/mbio.02446-22)
Supplement: FIG S2 [file mbio.02446-22-sf002.pdf]

Supplemental Figure 2

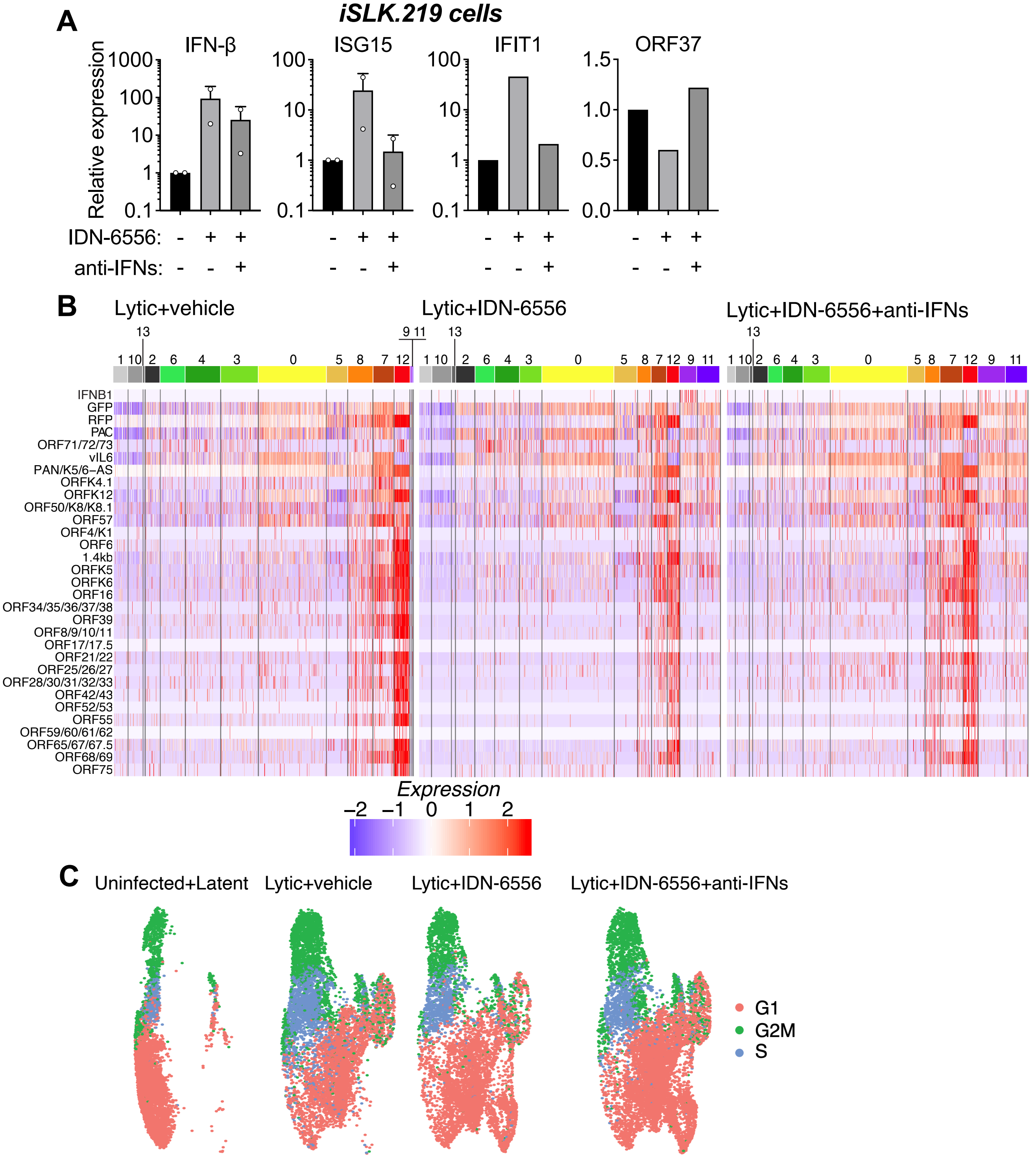

Supplemental Figure 2. scRNAseq controls and additional analyses. (A) iSLK.219 cells were treated with doxycycline (1 μg/ml) to reactivate the lytic cycle, as well as IDN-6556 and anti-IFN antibodies where indicated. mRNAs levels of IFN-β, the ISGs ISG15 and IFIT1, and the KSHV gene ORF37 were measured by RT-qPCR four days after doxycycline addition. (B-C) Analysis of data from the scRNA-Seq experiment presented in Fig. 3A. (B) Heatmaps of expression of KSHV genes in each cell in the three lytic samples, sorted by cluster. The legend below the heatmaps defines what expression level the colors represent (arbitrary units). (G) UMAP diagram of cell cycle stage classification of cells in the 4 samples.
