# Supplementary material for: Caspase-Mediated Regulation and Cellular Heterogeneity of the cGAS/STING Pathway in Kaposi’s Sarcoma-Associated Herpesvirus Infection
Source: mBio. 2022 Oct 18;13(6):e02446-22. doi: 10.1128/mbio.02446-22 (PMC9765453; doi:10.1128/mbio.02446-22)
Supplement: TABLE S1 [file mbio.02446-22-st001.pdf]

Table S1. Characteristics of scRNA-Seq datasets

	Uninfected + latent	Lytic + vehicle	Lytic + IDN-6556	Lytic + IDN-6556 + anti-IFNS
Total reads	142,704,565	138,925,899	154,624,957	160,218,965
Total cells	5,777	8,310	7,029	9,638
Median reads per cell	22,881	14,739.5	19,083	14,085
Total reads used in analysis	130,047,844	136,742,207	148,241,619	156,671,882
Cells used in analysis	5,406	7,879	6,695	9,159
