# Supplementary material for: Caspase-Mediated Regulation and Cellular Heterogeneity of the cGAS/STING Pathway in Kaposi’s Sarcoma-Associated Herpesvirus Infection
Source: mBio. 2022 Oct 18;13(6):e02446-22. doi: 10.1128/mbio.02446-22 (PMC9765453; doi:10.1128/mbio.02446-22)
Supplement: FIG S3 [file mbio.02446-22-sf003.pdf]

Supplemental Figure 3

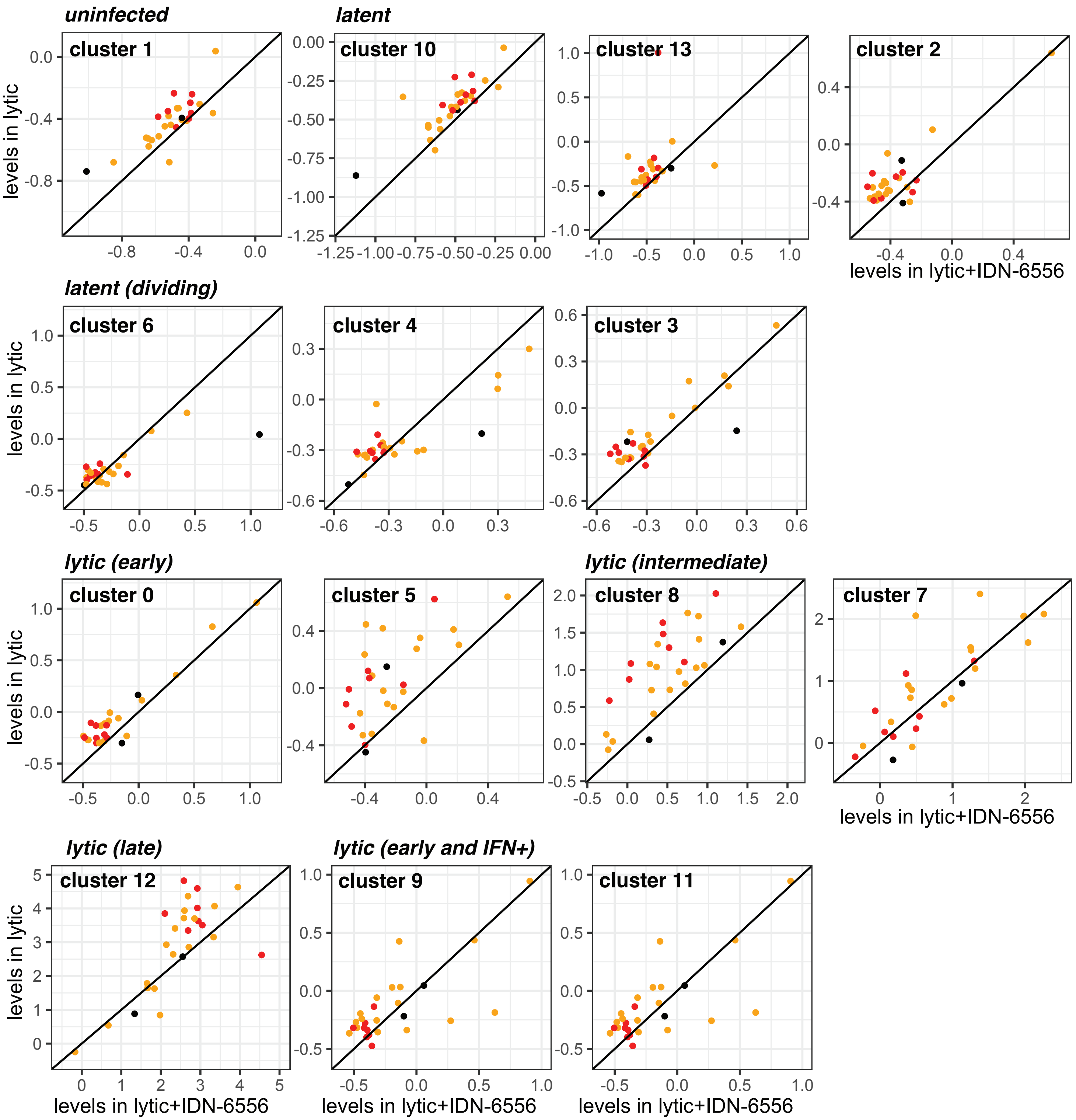

Supplemental Figure 3. Changes in viral gene expression between lytic iSLK.219 cells and lytic iSLK.219 cells treated with caspase inhibitors. Analysis of data from the scRNA-Seq experiment presented in Fig. 3A. Scatter plots show the average expression of viral genes in each of the clusters (ordered based on our classification) in iSLK.219 cells treated with doxycycline (lytic, y-axis) and iSLK.219 cells treated with doxycycline and caspase inhibitors (lytic + IDN-6556, x-axis). Black dots represent latent genes, yellow dots early genes and red dots late genes. The diagonal line represents equal levels.
