# Supplementary material for: Caspase-Mediated Regulation and Cellular Heterogeneity of the cGAS/STING Pathway in Kaposi’s Sarcoma-Associated Herpesvirus Infection
Source: mBio. 2022 Oct 18;13(6):e02446-22. doi: 10.1128/mbio.02446-22 (PMC9765453; doi:10.1128/mbio.02446-22)
Supplement: FIG S5 [file mbio.02446-22-sf005.pdf]

Supplemental Figure 5

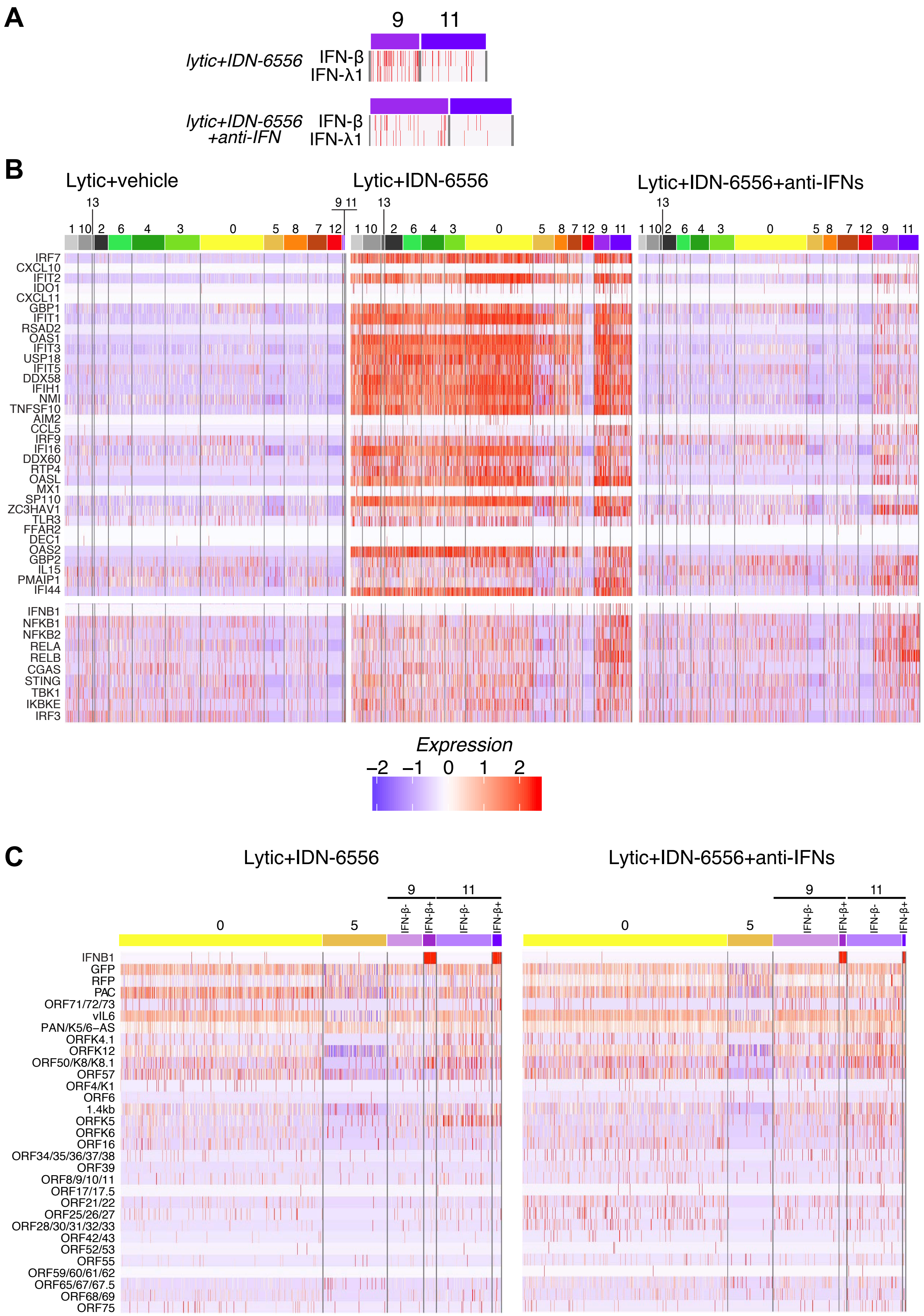

Supplemental Figure 5. Expression patterns of IFNs, ISGs and genes in the type I IFN induction pathway. Analysis of data from the scRNA-Seq experiment presented in Fig. 3A. (A) Heatmaps of expression of IFN-b and IFN-l1 in each cell in clusters 9 and 11 in the two IDN-6556 treated samples. (B) Heatmaps of expression of ISGs (top) and select genes from the type I IFN induction pathway (bottom) in each cell in the three lytic samples, sorted by cluster. (C) Heatmaps of viral gene expression in each cell in clusters 0, 5, 9, and 11, with cells in 9 and 11 divided by IFN-b status, in the indicated samples. The legend defines what expression level the colors represent (arbitrary units).
