# Supplementary material for: Caspase-Mediated Regulation and Cellular Heterogeneity of the cGAS/STING Pathway in Kaposi’s Sarcoma-Associated Herpesvirus Infection
Source: mBio. 2022 Oct 18;13(6):e02446-22. doi: 10.1128/mbio.02446-22 (PMC9765453; doi:10.1128/mbio.02446-22)
Supplement: TABLE S2 [file mbio.02446-22-st002.pdf]

Table S2. Number and percentage of cells in each cluster

Cluster	Classification	Dataset							
		Uninfected and latent		Lytic + vehicle		Lytic + IDN-6556		Lytic + IDN-6556 + anti-IFNs	
1	uninfected	2365	43.7%	378	4.8%	278	4.2%	251	2.7%
10	latent	16	0.3%	387	4.9%	439	6.6%	420	4.6%
13	latent	185	3.4%	40	0.5%	77	1.2%	79	0.9%
2	latent	1435	26.5%	390	4.9%	429	6.4%	457	5.0%
6	latent (dividing)	255	4.7%	663	8.4%	436	6.5%	437	4.8%
4	latent (dividing)	387	7.2%	942	12.0%	555	8.3%	624	6.8%
3	latent (dividing)	350	6.5%	1004	12.7%	499	7.5%	831	9.1%
0	lytic (early)	3	0.1%	1829	23.2%	1645	24.6%	2416	26.4%
5	lytic (early)	231	4.3%	559	7.1%	517	7.7%	523	5.7%
8	lytic (intermediate)	156	2.9%	667	8.5%	306	4.6%	453	4.9%
7	lytic (intermediate)	2	0.0%	555	7.0%	341	5.1%	693	7.6%
12	lytic (late)	0	0.0%	412	5.2%	274	4.1%	456	5.0%
9	lytic (early) and IFN+	7	0.1%	35	0.4%	385	5.8%	849	9.3%
11	lytic (early) and IFN+	14	0.3%	18	0.2%	514	7.7%	670	7.3%
	total	5406		7879		6695		9159	
